# The association between mammographic density and breast cancer risk in Chinese women: a systematic review and meta-analysis

**DOI:** 10.1186/s12905-024-02960-0

**Published:** 2024-02-20

**Authors:** Song Bai, Di Song, Ming Chen, Xiaoshu Lai, Jinfeng Xu, Fajin Dong

**Affiliations:** Department of Ultrasound, Shenzhen People’s Hospital (The Second Clinical Medical College, Jinan University; The First Affiliated Hospital, Southern University of Science and Technology), Shenzhen, Guangdong 518020 China

**Keywords:** Mammographic density, Breast cancer risk, Chinese women

## Abstract

**Purpose:**

Breast density has consistently been shown to be an independent risk factor for breast cancer in Western populations; however, few studies have evaluated this topic in Chinese women and there is not yet a unified view. This study investigated the association between mammographic density (MD) and breast cancer risk in Chinese women.

**Methods:**

The PubMed, Cochrane Library, Embase, and Wanfang databases were systematically searched in June 2023 to include all studies on the association between MD and breast cancer risk in Chinese women. A total of 13,977 breast cancer cases from 14 studies were chosen, including 10 case-control/cross-sectional studies, and 4 case-only studies. For case-control/cross-sectional studies, the odds ratios (ORs) of MD were combined using random effects models, and for case-only studies, relative odds ratios (RORs) were combinations of premenopausal versus postmenopausal breast cancer cases.

**Results:**

Women with BI-RADS density category II-IV in case-control/cross-sectional studies had a 0.93-fold (95% confidence interval [CI] 0.55, 1.57), 1.08-fold (95% CI 0.40, 2.94), and 1.24-fold (95% CI 0.42, 3.69) higher risk compared to women with the lowest density category. Combined RORs for premenopausal versus postmenopausal women in case-only studies were 3.84 (95% CI 2.92, 5.05), 22.65 (95% CI 7.21, 71.13), and 42.06 (95% CI 4.22, 419.52), respectively, for BI-RADS density category II-IV versus I.

**Conclusions:**

For Chinese women, breast cancer risk is weakly associated with MD; however, breast cancer risk is more strongly correlated with mammographic density in premenopausal women than postmenopausal women. Further research on the factors influencing MD in premenopausal women may provide meaningful insights into breast cancer prevention in China.

**Supplementary Information:**

The online version contains supplementary material available at 10.1186/s12905-024-02960-0.

## Introduction

Breast cancer is the most common tumour among Chinese women and it is also one of the leading causes of cancer death in females [1, 2]. Although the overall incidence of breast cancer in China is lower than that in Western nations, the incidence of breast cancer in China is growing at an annual rate of 3% to 4%, which is higher than that of Western countries and nearly twice the world average(1.9%) [3]. To reduce the burden and mortality caused by breast cancer, early diagnosis is essential [4–6].

In 1976, Wolfe first proposed that mammographic density was associated with breast cancer [7]. Since then, a large number of investigations have indicated that the higher the breast density is in Western women, the higher the risk of breast cancer. Some studies have shown that women with dense breasts have a 4-to 6-fold higher risk of developing breast cancer than women with nondense breasts [8]. Some scholars have also added breast density to risk prediction models, such as the TyrerCuzick and Gail models, which demonstrated that the new model combined with percentage density significantly improved the area under the subject working feature curve (AUC), which distinguishes more accurately between high-risk and low-risk groups [9, 10]. Thus, an accurate understanding of the function of breast density in the risk of breast cancer and combining it with the risk prediction model are highly valuable in establishing breast cancer screening and prevention strategies with different risk stratifications.

However, these studies have been performed mainly on Western women [11, 12]. There have been few studies on this area in large Chinese populations, and there is no consensus yet. It is questioned whether this increased risk ratio is also applicable to Chinese women. Thus, this study aimed to investigate and clarify the association between mammographic density (MD) and breast cancer risk in Chinese women.

## Materials and methods

This systematic review and meta-analysis was registered in PROSPERO (registration number CRD42021268523). Two independent reviewers (S.B. and D.S.) with comparable levels of experience conducted this study, including the processes of literature screening and quality assessment in accordance with the PRISMA guidelines [13]. Consensus between the two investigators or discussion with a third reviewer (XS.L.) was utilized to resolve disagreements. Interobserver consistency was evaluated by percentage agreement between the reviewers and Cohen’s kappa, see Supplementary material 1: Appendix A.

In this investigation, the researchers analysed publicly accessible information that was collected in a way that did not directly present patient identification. As a result, the investigation did not require informed consent or review board approval.

### Search strategy

The studies were searched using Medical Subject Headings (MeSH) and free words in the PubMed, Cochrane Library, Embase, and Wanfang databases up to June 2023. No language restrictions were applied. To ensure that other pertinent studies were covered, the references of all relevant papers and reviews were also reviewed. The search strategy is detailed in Supplementary material 1: Appendix B.

### Inclusion and exclusion criteria

The inclusion criteria for the articles were as follows:(1) research examined the relationship between Chinese women’s mammographic density and breast cancer risk; (2) observational studies, including cohort studies, cross-sectional studies, case-control studies, and case-only studies; (3) articles for which the full text was available and that contained original data; and (4) studies conducted only in mainland China. The most extensive study was chosen if multiple articles used the same study population. Review articles, editorials, letters, case reports, meeting abstracts, and duplicate studies were excluded.

### Study selection

Endnote was used to duplicate the identified articles. After the titles and abstracts were screened, the full texts were assessed according to the inclusion and exclusion criteria to select the final articles for analysis.

### Data collection

From each qualified article, the following information was extracted using a data extraction sheet: study information (first author’s name, publication year, region, study type), study population characteristics: number and age of breast cancer cases and controls (age refers to the whole population unless specified), type of breast cancer, variables adjusted, in case multiple methods categorizing MD were reported, the qualitative index was used. We collected the number of cases and controls in every density category from each individual study. If the pre-menopausal and post-menopausal groups were not clearly defined, then the age of 55 is used as the cut-off for menopause.

### Quality assessment

The methodological quality of the case-control,cross-sectional, and case-only studies was evaluated using the Newcastle-Ottawa Scale (NOS),the AHRQ (Agency for Healthcare Research and Quality) instrument, and the Methodological Index for Nonrandomized Studies (MINOS) instrument, (see Supplementary material 1: Appendix C-E); the full marks for the three instruments are nine, eleven, and eight scores, respectively. This quality assessment was performed independently by two reviewers (S.B. and D.S.) and the final results were based on consensus. Methodological deficiencies were defined as more than fifty percent of the studies not receiving a single star on this item.

### Statistical analysis

Crude OR/RORs were calculated from raw data provided in three types of studies. We extracted the number of breast cancer groups and/or control groups associated with different breast density categories. For case-control/cross-sectional studies, women with breast density BI-RADS II, III, or IV were respectively compared to women with BI-RADS density I by estimating pooled odds ratios (ORs) and related 95% confidence intervals (CIs). Similarly, ORs and related 95% CIs were calculated for comparisons of the BI-RADS density categories (I + II versus III + IV). In case-only studies, we firstly calculated the relative odds ratio (ROR) for each study, and then combined RORs and 95% CIs were calculated by conducting meta-analyses applying random effect models for two-category and four category comparisons. ROR = (number of premenopausal women with BI-RADS II, III or IV) * (number of postmenopausal women with BI-RADS I)/ (number of premenopausal women with BI-RADS I) * (number of postmenopausal women with BI-RADS II, III or IV) for four-category comparisons; for two-category comparisons, the ROR = (number of premenopausal women with BI-RADS III + IV) * (number of postmenopausal women with BI-RADS I + II)/ (number of premenopausal women with BI-RADS I) * (number of postmenopausal women with BI-RADS III + IV).

Heterogeneity was assessed by using the I^2^ statistic. Heterogeneity was considered to be present at either *p* < 0.05 or I^2^ > 50%. Random effects models were utilized if there was significant heterogeneity. Explanations for between-study heterogeneity (if any) in breast cancer risk were conducted by sensitivity analyses.

Publication bias was visually assessed using funnel plots and Egger tests. A *P* < 0.10 for funnel plots and *p* < 0.05/not containing 0 in 95% CI for Egger tests were considered to indicate the presence of publication bias. Stata version 15.0 was used for all analyses.

## Results

### Included studies

**Fig. 1:**
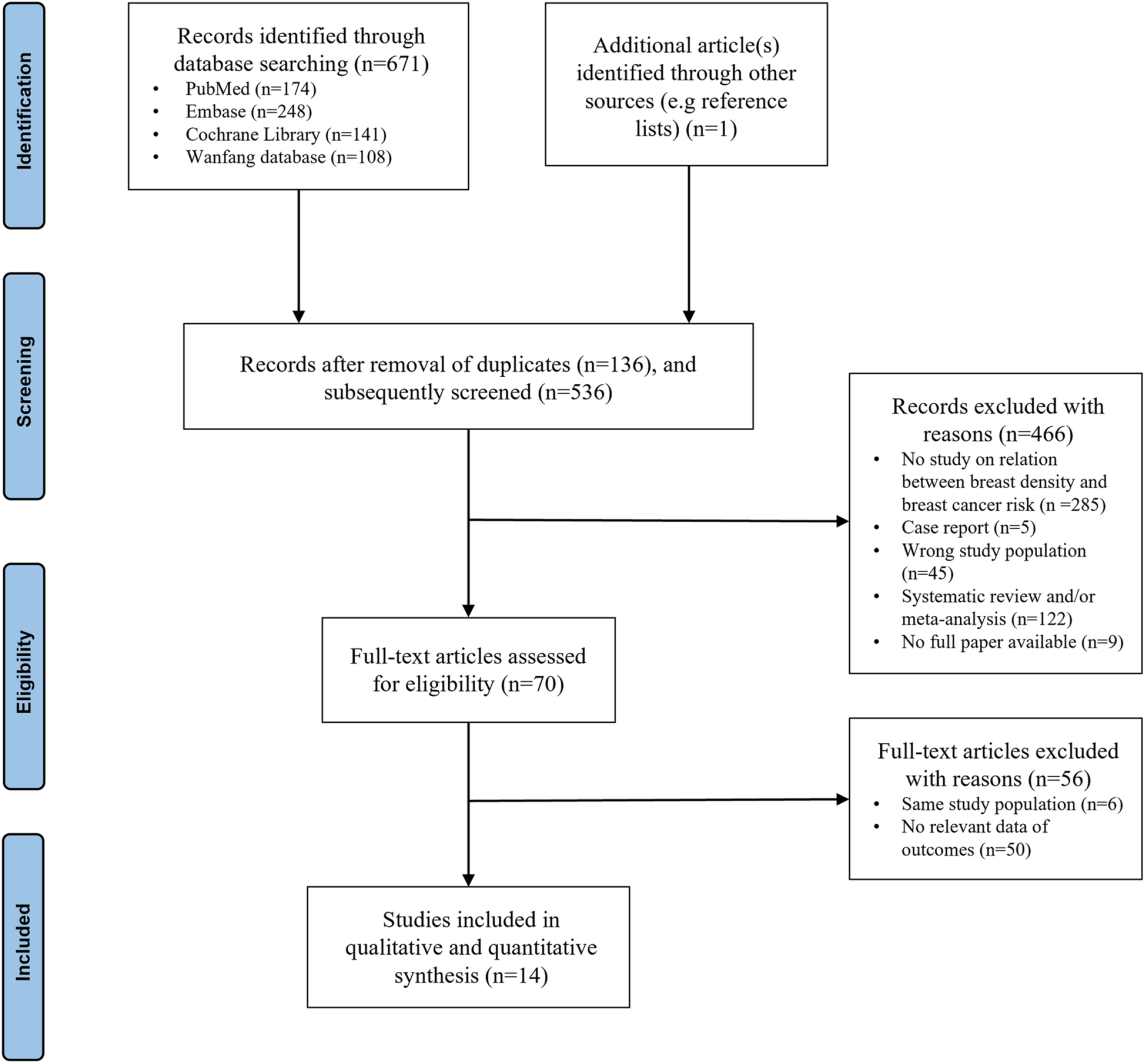
Flowchart of study selection

A total of 671 studies were identified after the screening (Fig. 1). By checking relevant articles’ references, an additional investigation was found [14]. After the elimination of 136 duplicates, 536 articles were screened on title and abstract and 466 articles were eliminated since they did not meet the inclusion criteria, leaving 70 papers of which full texts were thoroughly evaluated. Fourteen studies were eventually included in this systematic review and meta-analysis [14–27] after 56 articles were eliminated for the reasons shown in Fig. 1.

### Study characteristics

The main characteristics of the included studies in this meta-analysis are shown in Tables 1 and 2. With eight case-control studies [15, 17, 18, 20, 22, 24, 25, 27], two cross-sectional studies [16, 19], and four case-only studies [14, 21, 23, 26], the fourteen studies included in the systematic review and meta-analysis comprised a total of 64,367 Chinese women with 13,977 cases and 50,390 cancer-free women. Eleven studies defined breast cancer as invasive cancer or DCIS [15–20, 22, 24–27], and three included data for invasive breast cancer only [14, 21, 23]. All the studies defined control groups or cancer-free women as women who were not diagnosed with breast cancer [14–27]. All the studies used BI-RADS (ten used the 4th Edition [14–20, 22, 24, 27] and four used the 5th Edition [21, 23, 25, 26]) to subjectively assess breast density, and one of the studies also used the Quantra method to objectively examine breast density [25].

**Table 1:** Main characteristics of case-control/cross-sectional studies included in the meta-analysis

S.no.	First author, year (ref)	Study design	Region	Adj: variables adjusted	Type of BC Invasive or invasive and DCIS	Mammographic density categories [no/little, low, medium, high]	Age Mean (SD) or range	Controls (n)	Breast cancer cases (n)	Quality score by NOS/AHRQ
1	Du, 2009 [15]	Case-control	Beijing	Age	Both	BI-RADS^a^ [1, 2, 3, 4]	22 ~ 88	1559	62	6
2	Dai, 2014 [16]	Cross-sectional	Tianjin, Nanchang, Beijing, Shenyang	Age, BMI, study area, and other variables	Both	BI-RADS^a^ [1, 2, 3, 4]	45 ~ 65	28,302	86	10
3	Chen, 2015 [17]	Case-control	Harbin	Not formally age-adjusted	Both	BI-RADS^a^ [1, 2, 3, 4]	Cannot retrieve data but a wide range	382	105	7
4	Wu, 2015 [18]	Case-control	Kunshan	Age	Both	BI-RADS^a^ [1, 2, 3, 4]	19 ~ 82	4102	139 31:26^c^ (premenopausal: postmenopausal)	6
5	Yang, 2016 [19]	Cross-sectional	Guangzhou	BMI, age, duration of breastfeeding, and other variables	Both	BI-RADS^a^ [1, 2, 3, 4]	27 ~ 57(premenopausal)	1689	10	8
6	Yu, 2016 [20]	Case-control	Cangzhou	Age	Both	BI-RADS^a^ [1, 2, 3, 4]	22 ~ 85	4822	184 128:56 (premenopausal: postmenopausal)	7
7	Du, 2019 [22]	Case-control	Harbin	Age, menopausal status, and breastfeeding	Both	BI-RADS^a^ [1, 2, 3, 4]	Cannot retrieve data but a wide range	588	237 131:106 (premenopausal: postmenopausal)	7
8	Ye, 2019 [24]	Case-control	Shenyang	Age, menopausal status, age at menopause, and other variables	Both	BI-RADS^a^ [1, 2, 3, 4]	57 (5.1)	2284	735	8
9	Wang, 2020 [25]	Case-control	Taiyuan	BMI, menstrual status, and other variables	Both	BI-RADS^b^ [1, 2, 3, 4] Quantra classification [1, 2, 3, 4]	26 ~ 81	932	466	7
10	Xu, 2021 [27]	Case-control	Ningbo	Age, menopausal status, and other variables	Both	BI-RADS^a^ [1, 2, 3, 4]	Cannot retrieve data but a wide range	5730	270	8

*BMI* Body mass index, *SD* Standard deviation, *DCIS* Ductal carcinoma in situ, *BC* Breast cancer

^a^ACR BI-RADS Atlas 4th edition [28]

^b^ACR BI-RADS Atlas 5th edition [29]

^c^The 40–60 years age group in the study was not included in the pre- or post-menopausal group

**Table 2:** Main characteristics of case-only studies included in the meta-analysis

S.no	First author, year (ref)	Study design	Region	Adj: variables adjusted	Type of BC Invasive or invasive and DCIS	Mammographic density categories [no/little, low, medium, high]	Age at breast cancer diagnosis Mean (SD) or range	Breast cancer cases (n)	Quality score by MINOS
1	Li, 2017 [21]	Retrospective	Kunming	Age, menopausal status, and other variables.	Invasive breast cancer only	BI-RADS^b^ [1 + 2,3 + 4]	22 ~ 69	69 41:28 (premenopausal: postmenopausal)	6
2	Li, 2019 [23]	Retrospective	Beijing	Age, BMI, menopausal status, parity, and other variables.	Invasive breast cancer only	BI-RADS^b^ [1 + 2,3,4]	51.7 (10.7)	1779 889:890 (premenopausal: postmenopausal)	7
3	Zhao, 2020 [14]	Retrospective	Harbin	Not formally age-adjusted.	Invasive breast cancer only	BI-RADS^a^ [1 + 2,3 + 4]	25 ~ 69 47 (NK)	119 68:51 (premenopausal: postmenopausal)	6
4	Ji, 2021 [26]	Retrospective	Tianjin	Age.	Both	BI-RADS^b^ [1, 2, 3, 4]	19 ~ 93 54.3 (11.2)	9716 4892:4824 (premenopausal: postmenopausal)	6

*BMI* Body mass index, *SD* Standard deviation, *DCIS* Ductal carcinoma in situ, *BC* Breast cancer

^a^ACR BI-RADS Atlas 4th edition [28]

^b^ACR BI-RADS Atlas 5th edition [29]

All studies reported crude data (the frequency of cases corresponding to different breast density categories in the control group and breast cancer group). In addition, twelve studies adjusted for the most important confounding factor, age/BMI [15, 16, 18–27], four studies adjusted for only age [15, 18, 20, 26], and the other eight studies adjusted for two or more covariates [16, 19, 21–25, 27].

### Quality assessment

For case–control studies, the quality assessment scores ranged from 6–8. According to the NOS, methodological deficiencies are mainly involved [1]: no clear description of the nonresponse rate (87.5%); [2]: inadequate case definitions (62.5%); and [3]: no control for confounding factors (50.0%).

For cross-sectional studies, the scores ranged from 8–10. According to the AHRQ, methodological deficiencies are mainly involved [1]: no clear description of follow-up results (100%); [2]: no explanation of how missing data is handled in the analysis (50.0%).

For case-only studies, the scores ranged from 6–7. According to the MINOS, most methodological deficiencies are mainly involved [1]: no clear description of follow-up results (100%); [2]: no blinding was performed (75%).

### Results of the meta-analysis

#### For case-control and cross-sectional studies

**Fig. 2:**
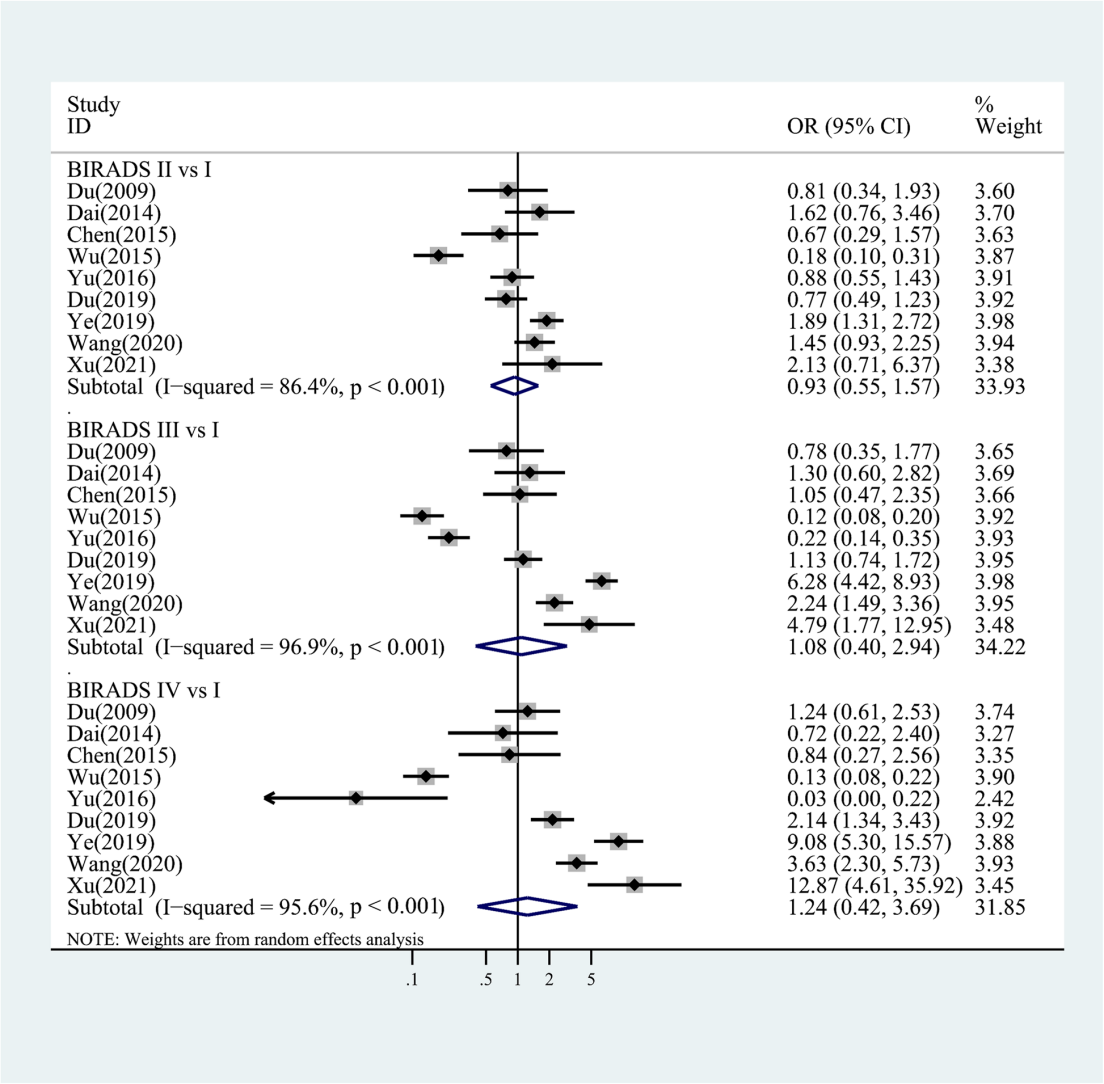
Case-control/cross-sectional studies: study-specific and random effects combined estimates for breast cancer associated with four categories of MD. OR, odds ratio; CI, confidence interval

**Fig. 3:**
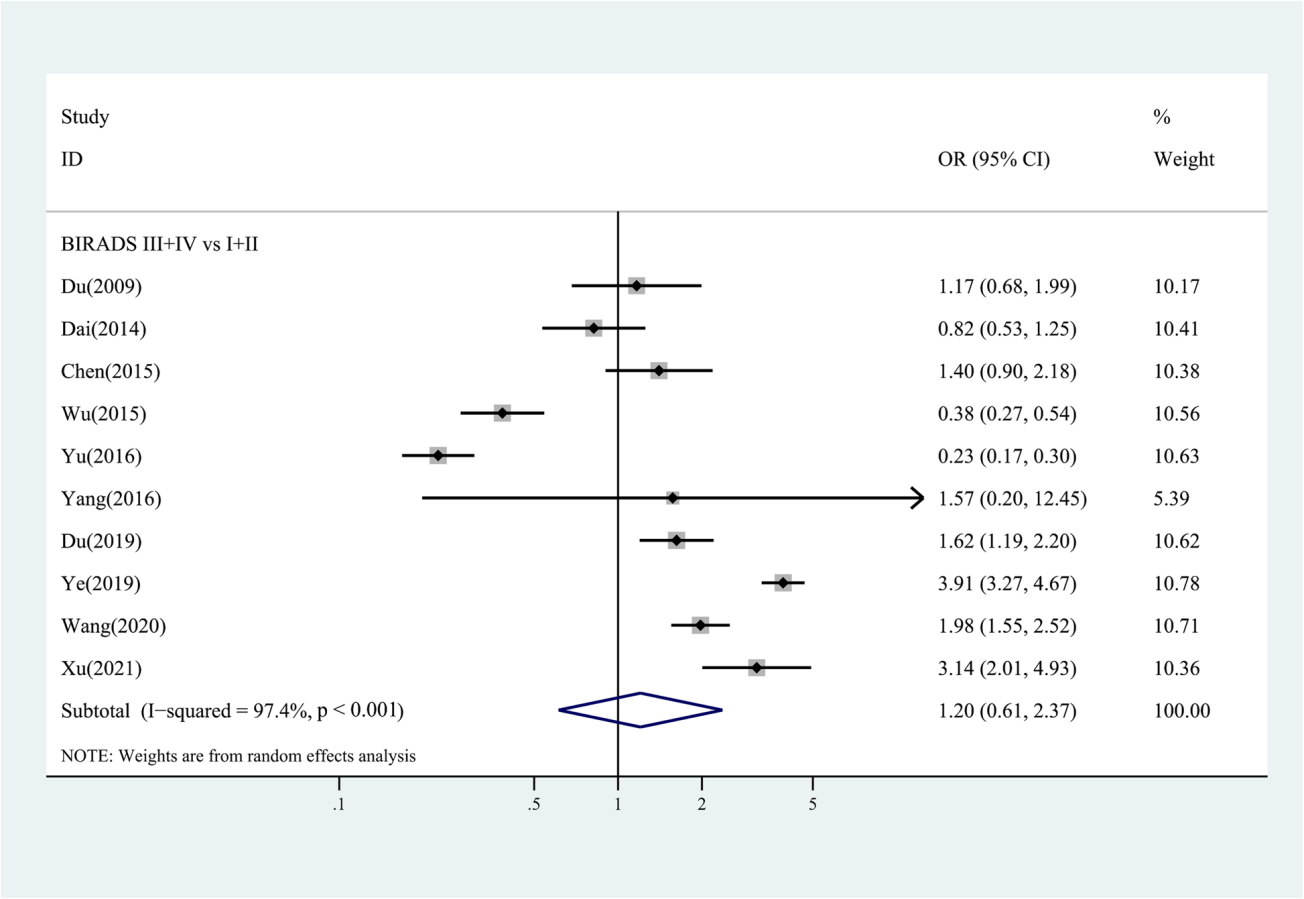
Case-control/cross-sectional studies: study-specific and random effects combined estimates for breast cancer associated with two categories of MD. OR, odds ratio; CI, confidence interval

Figure 2 shows four-category data of the change trends in breast cancer risk as breast density increases. For Chinese women with BI-RADS II, III and IV compared to women with BI-RADS I, pooled ORs of 0.93 (95% CI 0.55,1.57; I^2^ = 86.4%, *p* < 0.001), 1.08 (95% CI 0.40,2.94; I^2^ = 96.9%, *p* < 0.001) and 1.24 (95% CI 0.42,3.69; I^2^ = 95.6%, *p* < 0.001) were found. Figure 3 shows two-category data, for women with BI-RADS III + IV compared to women with BI-RADS I + II, the pooled OR of 1.20 (95% CI 0.61,2,37; I^2^ = 97.4%, *p* < 0.001) was found.

#### For case-only studies

**Fig. 4:**
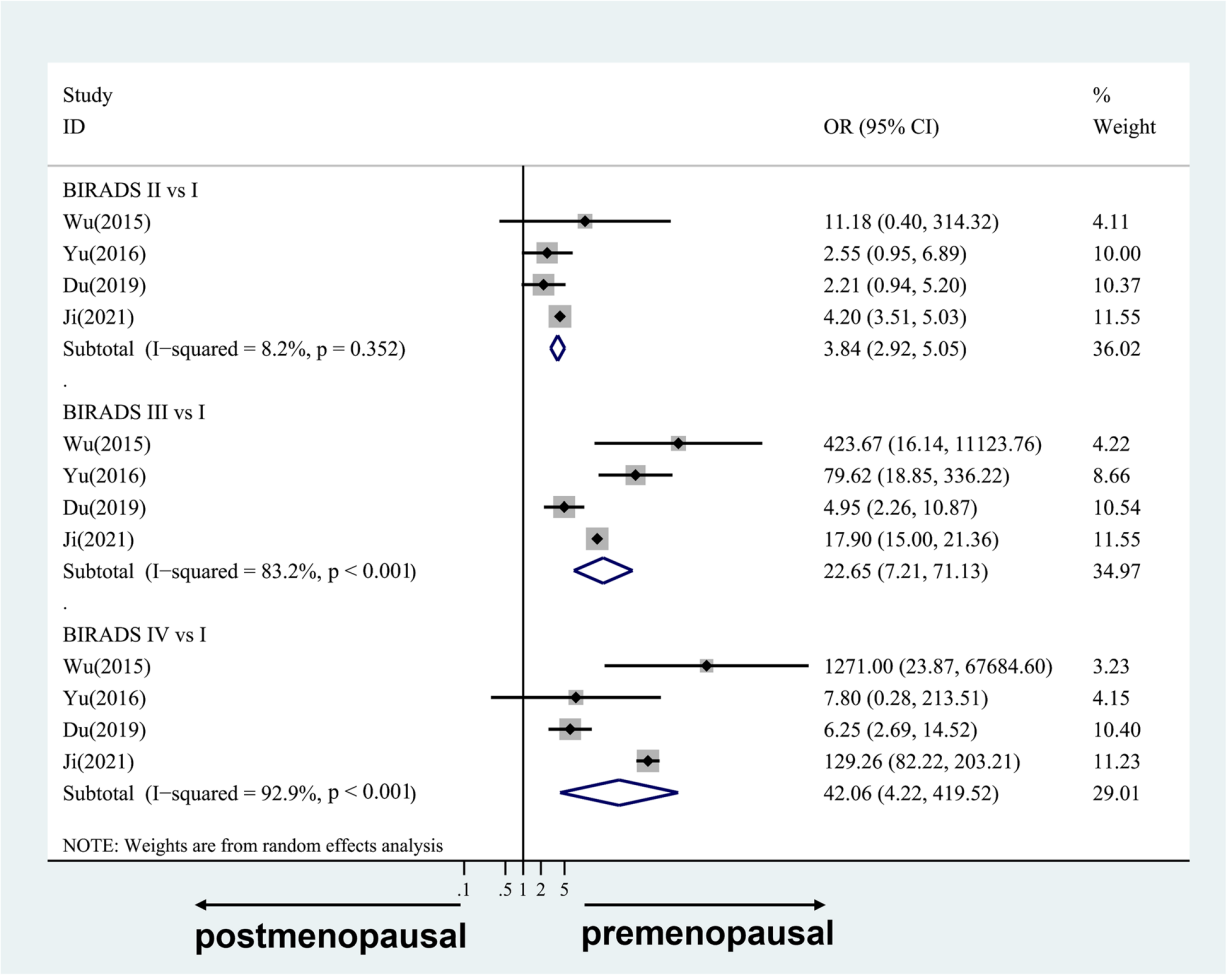
Case-only studies: study-specific and random effects combined relative risks of four-category MD associated with premenopausal versus postmenopausal breast cancer cases. OR, odds ratio; CI, confidence interval

**Fig. 5:**
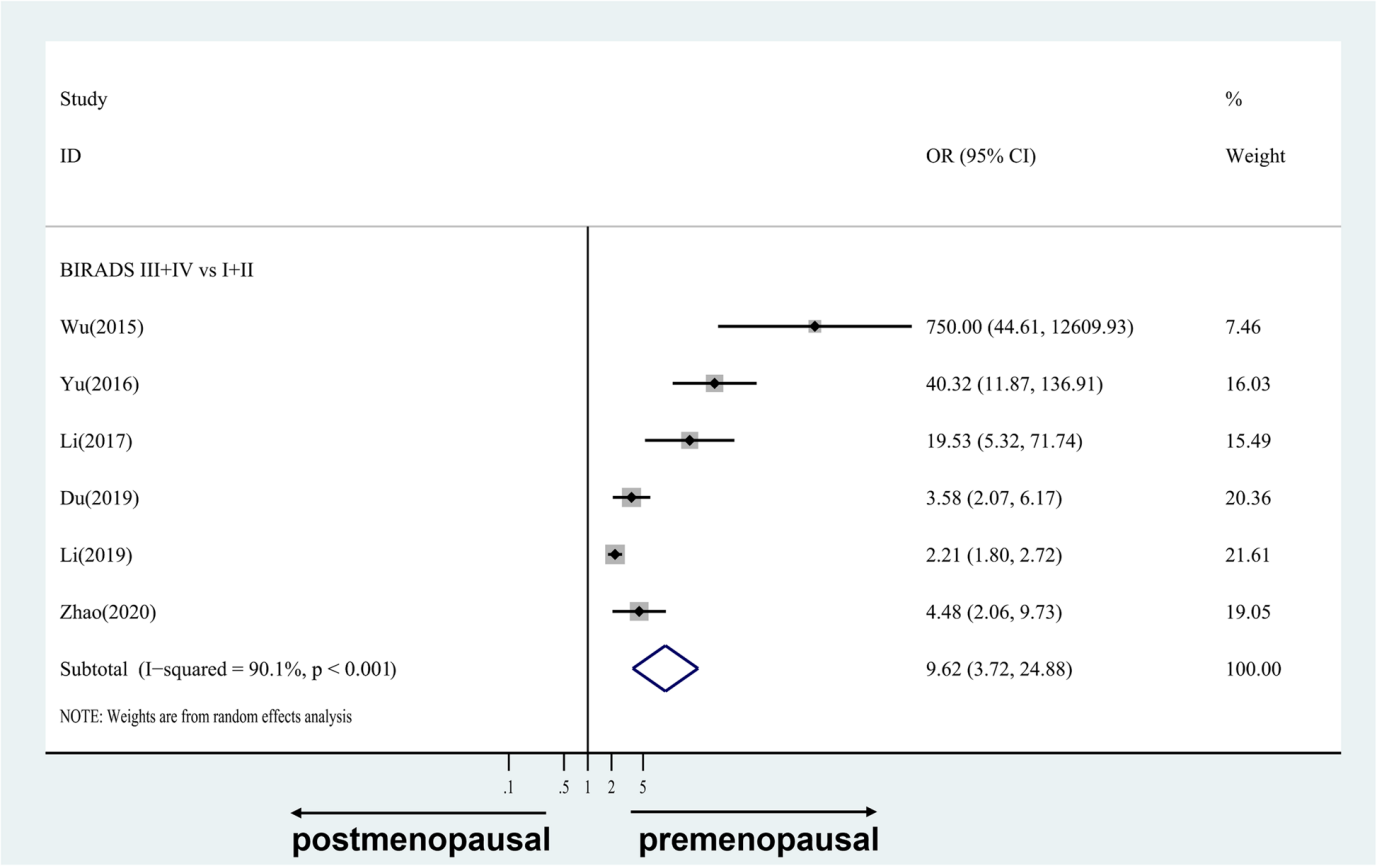
Case-only studies: study-specific and random effects combined relative risks of two-category MD associated with premenopausal versus postmenopausal breast cancer cases. OR, odds ratio; CI, confidence interval

Figure 4 shows the four-category data, while Fig. 5 shows the two-category data. The estimates to the right of the null ROR = 1 line demonstrate that premenopausal status was more strongly related to MD than was postmenopausal status for Chinese women. There was a strong difference in premenopausal status compared with postmenopausal status, with combined RORs for BI-RADS II, III, and IV versus I of 3.84 (95% CI 2.92, 5.05; I^2^ = 8.2%, *p* = 0.352), 22.65 (95% CI 7.21,71.13; I^2^ = 83.2%, *p* < 0.001), and 42.06(95% CI 4.22,419.52; I^2^ = 92.9%, *p* < 0.001), respectively. Similar results were found for the two-category data, in which the combined ROR for BI-RADS III + IV versus I + II was 9.62(95% CI 3.72,24.88; I^2^ = 90.1%, *p* < 0.001).

However, heterogeneity was observed according to the above heterogeneity criteria. Sensitivity analyses were performed to explore between-study heterogeneity. According to the sensitivity analysis, there were no significant changes in the pooled ORs in any of the comparison categories. All the sensitivity analyses are presented in Supplementary material 1: Appendix F, Supplementary Table 1.

### Publication bias

The funnel plots of the fourteen included studies are shown in Supplementary material 1: Appendix G, Supplementary Figs. 1-8. There was no indication of significant publication bias in general except for BI-RADS III+IV versus I+II for case-only studies (Supplementary Fig. 8). Egger’s tests showed the same results (Supplementary material 1: Appendix H, Supplementary Table 2).

## Discussion

This systematic review and meta-analysis investigated fourteen observational studies that evaluated the relationship between mammographic density and breast cancer risk in Chinese women. In our study, breast cancer risk did not increase markedly with increasing breast density for Chinese women, and having BI-RADS density II-IV resulted in a 0.93-fold, 1.08-fold, and 1.24-fold higher breast cancer risk than did having BI-RADS I. Breast cancer risk was also found more strongly linked with mammographic density in premenopausal women than in postmenopausal women.

Compared with the strong linear trends identified by previous studies for Western women, a weak association between MD and breast cancer risk was found in Chinese women. This conclusion is different from that of previous papers that included all races [8, 30]. In recent years, an increasing number of studies have focused on the factors related to breast density and the incidence of breast cancer and their interactions, and the differences in these factors between China and the West may directly or indirectly lead to differences in the relationship between MD and breast cancer risk. First, as a highly heterogeneous malignant tumour, breast cancer has certain differences in the occurrence and development among different ethnic groups. According to previous literature, although the incidence of breast cancer in China is increasing rapidly, it is still much lower than that in Western populations [31]. In addition, there are some differences in the distribution of breast density among women of different races. It has been reported that Chinese women have higher percentage mammographic density (PD) and dense area (DA) than Australian women [32]. Thus, Chinese women have both denser mammary glands and a lower incidence of breast cancer than European and American women. Therefore, it is believed that the difference between MD and breast cancer risk in Chinese and Western women may be related to ethnic differences to some extent. Another factor may be the different preferences for oestrogen replacement therapy between Chinese and Western women. The use of oestrogen replacement therapy in postmenopausal Chinese women is reportedly significantly lower than that in Western women [33, 34]. Postmenopausal hormone replacement therapy has been shown to increase breast gland density in postmenopausal women [35, 36]. Therefore, the history of estrogen replacement therapy may be one of the reasons why the postmenopausal breast density of European and American women exceeds that of Chinese women.

Another important factor may be obesity. Studies have shown that obesity can increase the risk of breast cancer by increasing oestrogen levels in the body [37]. However, obesity is negatively correlated with the percentage of breast gland density [38]. Previous studies have shown that the overall BMI of Chinese women is lower than that of Western women [39], but the percentage of breast density of Chinese women is higher [32]. The inverse effects of breast density percentage and low BMI on the risk of breast cancer coexist in Chinese women, which may lead to the poor ability of breast density percentage in predicting the risk of breast cancer.

In addition to the above factors, factors such as no history of childbearing, and late age at first childbirth (> 35 years old) in European and American populations have also been found to affect the risk of breast cancer by increasing breast density [40]. However, the epidemiological evidence of this aspect in China is not comprehensive. The influence of fertility pattern factors on MD and the incidence of breast cancer needs more research and exploration.

In this study, we also found that the effect of breast density on the risk of breast cancer was more obvious in premenopausal women. This may be due to the following mechanisms. Before menopause, breast density gradually decreases with age, and the decrease is most obvious during perimenopause [12]. Collagen in high-density breast tissue has a faster conversion rate than that in low-density breast tissue [41]. Dense breast tissue contains more collagen, a high concentration of insulin-like growth factor (IGF-1), and tissue inhibitor of metalloproteinase 3(TIMP-3). IGF-1 can not only stimulate the growth of breast epithelial cells and fibrositis components but also interact with oestrogen and increase the aggressiveness of breast cancer cells [42], thus increasing the risk of breast cancer in premenopausal women.

Due to the significantly earlier age of onset, a considerable proportion of breast cancer patients in China are premenopausal [43]. In addition, studies have demonstrated that menopause is associated with a decrease in mammographic density [44]. Therefore, the factors affecting the density of premenopausal women have significant public health implications for the prevention of breast cancer in China. This is consistent with the findings of other research on the entire population [45, 46].

This systematic review has several limitations. First, between-study heterogeneity existed in several analyses. Subgroup analyses were conducted to explain the reasons for heterogeneity and showed homogenous risk estimates. Second, there are differences in opinion regarding whether DCIS should be excluded in breast cancer cases. Third, in our systematic review, we mostly extracted raw data to calculate ORs/RORs because there are no available adjusted ORs from most of the literature. However, the baseline data in the paper were basically the same so as not to cause significant bias. Further limitations include density misclassification and recall bias of baseline data. The advantage of this article is that our retrieval had no language restrictions, and publication bias is unlikely. Another advantage was the relatively large number of included studies and sufficient women’s data were analyzed.

## Conclusion

This systematic review provides clear evidence that breast cancer risk is weakly associated with MD for Chinese women. Conclusions of studies on European and American women cannot be applied to Chinese women. However, the association between breast density and breast cancer risk is more prominent in younger premenopausal women. Observation of breast density at different ages, especially during perimenopause and premenopause, should be one of the main steps of breast cancer risk assessment in breast screening.

### Supplementary Information

**Supplementary material 1.**

## Data Availability

If someone wants to request the data from this study, please contact Song Bai (baisong@szhospital.com).
