# Supplementary material for: The association between mammographic density and breast cancer risk in Chinese women: a systematic review and meta-analysis
Source: BMC Womens Health. 2024 Feb 20;24:131. doi: 10.1186/s12905-024-02960-0 (PMC10877813; doi:10.1186/s12905-024-02960-0)
Supplement: Supplementary file 1 — Supplementary material 1. [file 12905_2024_2960_MOESM1_ESM.docx]

# **Appendix A-Reviewer Agreement**

|  | **Overall agreement** | **Cohen’s kappa** |
| --- | --- | --- |
| **Literature screening** |  |  |
| Screening on title and abstract | 96.6% | 0.854 |
| Screening on full text | 97.1% | 0.906 |
| **Quality assessment** |  |  |
| For case-control studies | 75% | 0.600 |
| For case-only studies | 75% | 0.500 |
| For cross-sectional studies | 50.0% | 0.333 |

# **Appendix B-Search Strategy**

**PubMed**

((Asian Continental Ancestry Group[MeSH Terms]) OR (China[Text Word]) OR (Chinese[Text Word])) AND ((Breast Density [MeSH Terms]) OR (mammographic breast density [Title/Abstract]) OR (breast density, mammographic[Title/Abstract]) OR (mammographic density [Title/Abstract]) OR (parenchymal pattern [Title/Abstract]) OR (Wolfe [Title/Abstract]) OR(BIRADS [Title/Abstract]) OR (Tabar [Title/Abstract]) OR (mammographic pattern [Title/Abstract]) OR (breast density [Title/Abstract])) AND ((Neoplasms [MeSH Terms]) OR (Tumor [Title/Abstract]) OR (Cancer [Title/Abstract]) OR (Malignancy [Title/Abstract]) OR (Malignant Neoplasm[Title/Abstract]) OR (Neoplasm, Malignant [Title/Abstract])).

**Embase**

(('Asian Continental Ancestry Group'/exp) OR (China OR Chinese):ab,ti) AND (('Breast Density'/exp) OR (mammographic breast density OR breast density, mammographic OR mammographic density OR parenchymal pattern OR Wolfe OR BIRADS OR Tabar OR mammographic pattern OR breast density):ab,ti) AND (('Neoplasms'/exp) OR (Tumor OR Cancer OR Malignancy OR Malignant Neoplasm OR Neoplasm, Malignant) :ab,ti).

**Cochrane**

(Asian people):ti,ab,kw OR ((China) OR (Chinese)) AND (Breast Density):ti,ab,kw OR ((mammographic breast density) OR (breast density, mammographic) OR (mammographic density) OR (parenchymal pattern) OR (Wolfe) OR(BIRADS) OR (Tabar) OR (mammographic pattern) OR (breast density)) AND (Neoplasms):ti,ab,kw OR ((Tumor) OR (Cancer) OR (Malignancy) OR (Malignant Neoplasm) OR (Neoplasm, Malignant))

**Wanfang database**

Chinese characters "breast cancer AND breast density" in the Chinese interface of Wanfang database were input to conduct the screening.

Since it is a local Chinese database, we did not add the search term "China", so as to avoid omissions as much as possible.

# **Appendix C-Newcastle-Ottawa Scale (NOS) quality assessment**

## **Case-control Studies**

Note: A study can be awarded a maximum of one star for each numbered item within the Selection and Exposure categories. A maximum of two stars can be given for Comparability.

*Selection*

1) Is the case definition adequate?

a) Yes, with independent validation (e.g. reference to primary record source such as medical record) **★**

b) Yes, e.g. record linkage (e.g. ICD codes in database) or based on self-reports with no reference to primary record

c) No description

2) Representativeness of the cases

a) Consecutive or obviously representative series of cases **★**

b) Potential for selection biases or not stated

3) Selection of Controls

a) Community controls **★**

b) Hospital controls

c) No description

4) Definition of Controls

a) No history of disease (Breast cancer as endpoint) **★**

b) No description of source/No mention of history of outcome

*Comparability*

1) Comparability of cases and controls on the basis of the design or analysis

a) Study controls for age **★**

b) Study controls for any additional factor, such as Body Mass Index (BMI), parity, menopausal status**★**

*Exposure*

1) Ascertainment of exposure

a) Secure record (e.g. surgical records) **★**

b) Structured interview/assessment of exposure where blind to case/control status **★**

c) Ascertainment of exposure not blinded to case/control status

d) Written self-report or medical record only

e) No description

2) Same method of ascertainment for cases and controls

a) Yes **★**

b) No

3) Non-Response rate

a) Same rate for both groups **★**

b) Non respondents described

c) Rate different and no designation

d)Not stated

# **Appendix D–AHRQ (Agency for Healthcare Research and Quality) instrument**

## **Cross-sectional Studies**

Note: A study can be awarded a single star for each numbered item, the total score is 11 stars.

| Items | Judgment |
| --- | --- |
| 1、Define the source of information (survey, record review) | Yes**★**/No/Unclear |
| 2、List inclusion and exclusion criteria for exposed and unexposed subjects (cases and controls) or refer to previous publications | Yes**★**/No/Unclear |
| 3、Indicate time period used for identifying patients | Yes**★**/No/Unclear |
| 4、Indicate whether or not subjects were consecutive if not population-based | Yes**★**/No/Unclear |
| 5、Indicate if evaluators of subjective components of study were masked to other aspects of the status of the participants | Yes**★**/No/Unclear |
| 6、Describe any assessments undertaken for quality assurance purposes (e.g., test/retest of primary outcome measurements) | Yes**★**/No/Unclear |
| 7、Explain any patient exclusions from analysis | Yes**★**/No/Unclear |
| 8、Describe how confounding was assessed and/or controlled | Yes**★**/No/Unclear |
| 9、If applicable, explain how missing data were handled in the analysis | Yes**★**/No/Unclear |
| 10、Summarize patient response rates and completeness of data collection | Yes**★**/No/Unclear |
| 11、Clarify what follow-up, if any, was expected and the percentage of patients for which incomplete data or follow-up was obtained | Yes**★**/No/Unclear |

# **Appendix E-MINOS (Methodological Index for Non-randomized Studies) instrument**

## **Case-only Studies**

Note: A study can be awarded a single star for each numbered item, the total score is 8 stars.

| **Items** | **Prompt** |
| --- | --- |
| 1、The research objective is given clearly**★** | The problem defined should be precise and relevant to the literature available. |
| 2、Patient coherence**★** | All potential patients (who met the criteria for admission) were enrolled during the study (no exclusions or reasons for exclusion are given). |
| 3、Collection of anticipated data**★** | Data were collected according to the study protocol developed before the start of the study. |
| 4、The endpoints can properly reflect the purpose of the study**★** | Clearly explain the criteria used to evaluate outcome indicators that are consistent with the defined problem. At the same time, endpoints should be evaluated on the basis of intention-to-treat analysis. |
| 5、The objectivity of the evaluation of endpoints**★** | The objective endpoints were evaluated by the evaluator single-blind method, and the subjective endpoints were evaluated by the evaluator double-blind method. Otherwise, reasons should be given for not performing a blind evaluation. |
| 6、Whether the follow-up time is sufficient**★** | Follow-up should be long enough to allow evaluation of endpoints and possible adverse events. |
| 7、The loss of follow-up rate is less than 5%**★** | All patients should be followed up. Otherwise, the proportion of lost follow-up cannot exceed the proportion of patients reflecting the primary endpoint. |
| 8、Whether the sample size was estimated**★** | According to the incidence of expected outcome events, the sample size and 95% confidence interval for different study outcomes were calculated. The information provided was able to compare the expected results with the actual results in terms of statistically significant differences and estimated confidence levels. |

# **Appendix F**

**Supplementary Table 1** Results of sensitivity analyses

Note: There were no significant changes for the pooled ORs in any of the comparison categories.

|  | **Pooled OR** | **95%CI** |
| --- | --- | --- |
| **For case-control/cross-sectional studies**  **sectional**  Elimination of Ji2021  Elimination of Ji2021  **studies** |  |  |
| **BI-RADS II vs I**  Elimination of Wu2015^18^  Elimination of Ye2019^24^ | 1.17  0.84 | 0.86-1.60  0.49-1.45 |
| **BI-RADS III vs I**  Elimination of Wu2015^18^  Elimination of Yu2016^20^  Elimination of Ye2019^24^ | 1.43  1.33  0.85 | 0.59-3.46  0.49-3.59  0.35-2.09 |
| **BI-RADS IV vs I**  Elimination of Wu2015^18^ | 1.85 | 0.83-4.15 |
| **BI-RADS III+IV vs I+II**  Elimination of Wu2015^18^  Elimination of Yu2016^20^  Elimination of Ye2019^24^ | 1.38  1.47  1.04 | 0.70-2.73  0.85-2.54  0.55-1.98 |
| **For case-only studies** |  |  |
| **BI-RADS III vs I**  Elimination of Ji2021^26^ | 40.11 | 3.15-510.70 |
| **BI-RADS IV vs I**  Elimination of Du2019^22^  Elimination of Ji2021^26^  **BI-RADS III+IV vs I+II**  Elimination of Li2019^23^ | 102.32  25.08  15.91 | 13.61-769.51  1.42-442.86  4.55-55.64 |

# **Appendix G**


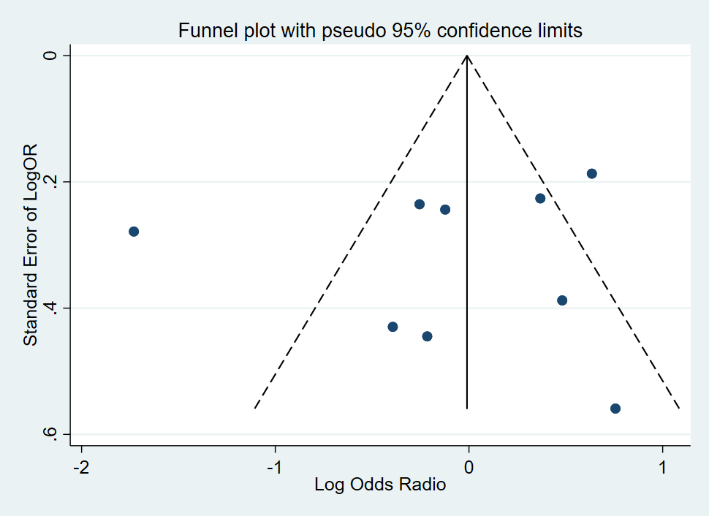


**Supplementary Fig.1:** Funnel plot corresponding to BI-RADS II vs I in the forest plot of Fig.2.


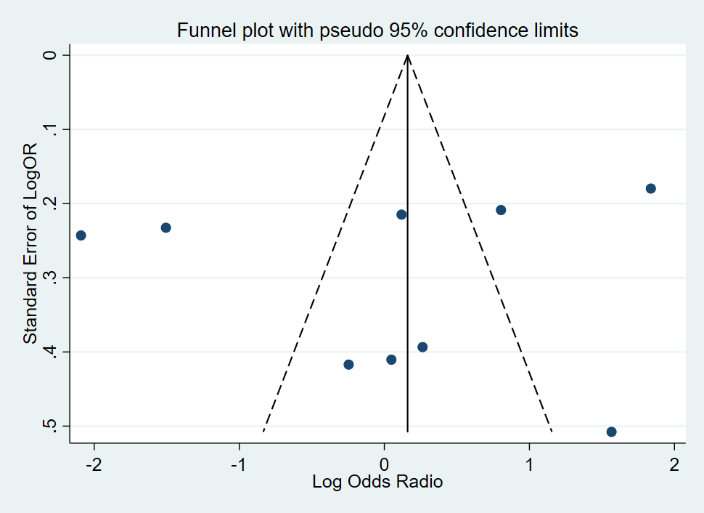


**Supplementary Fig.2:** Funnel plot corresponding to BI-RADS III vs I in the forest plot of Fig.2.


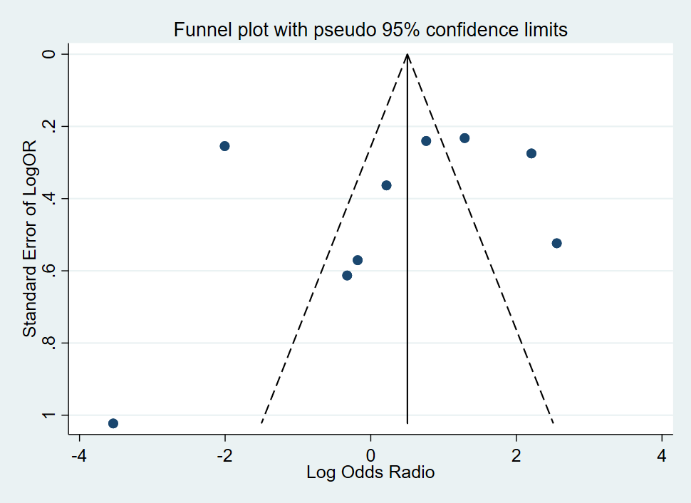


**Supplementary Fig.3:** Funnel plot corresponding to BI-RADS IV vs I in the forest plot of Fig.2.


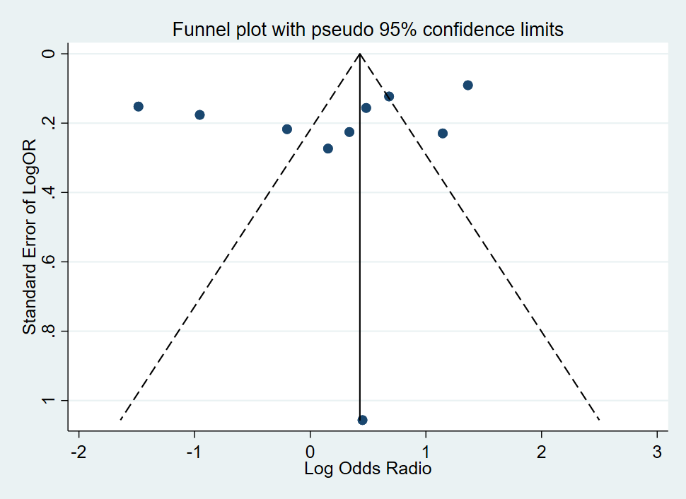


**Supplementary Fig.4:** Funnel plot corresponding to BI-RADS III+IV vs I+II in the forest plot of Fig.3.


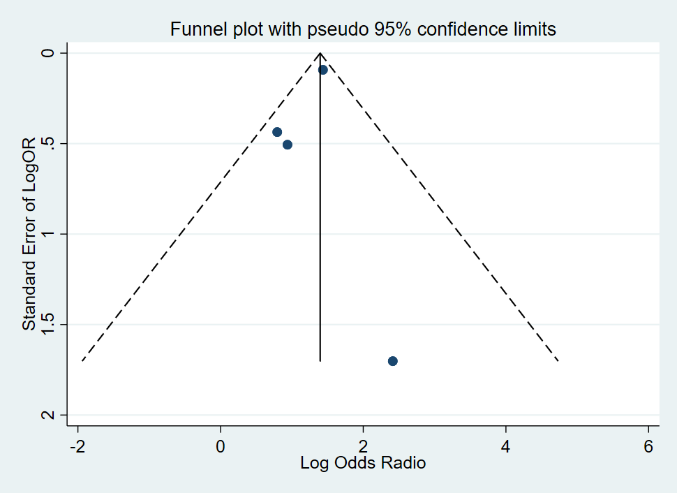


**Supplementary Fig.5:** Funnel plot corresponding to BI-RADS II vs I in the forest plot of Fig.4.


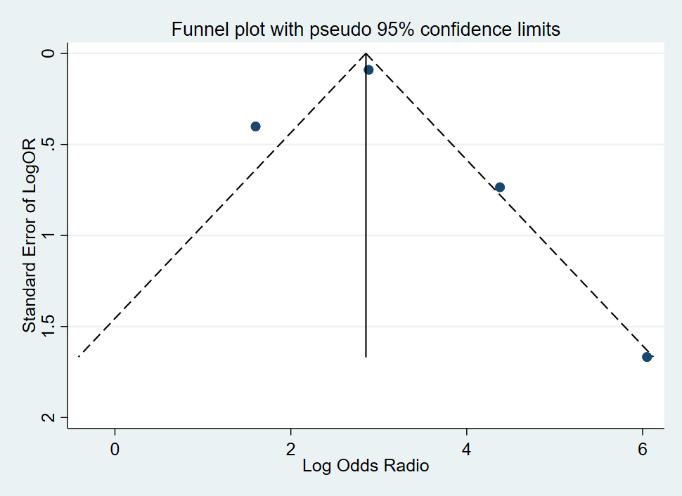


**Supplementary Fig.6:** Funnel plot corresponding to BI-RADS III vs I in the forest plot of Fig.4.


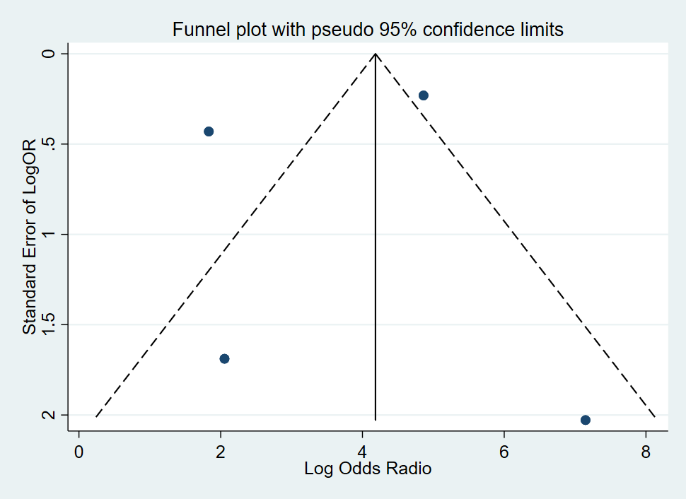


**Supplementary Fig.7:** Funnel plot corresponding to BI-RADS IV vs I in the forest plot of Fig.4.


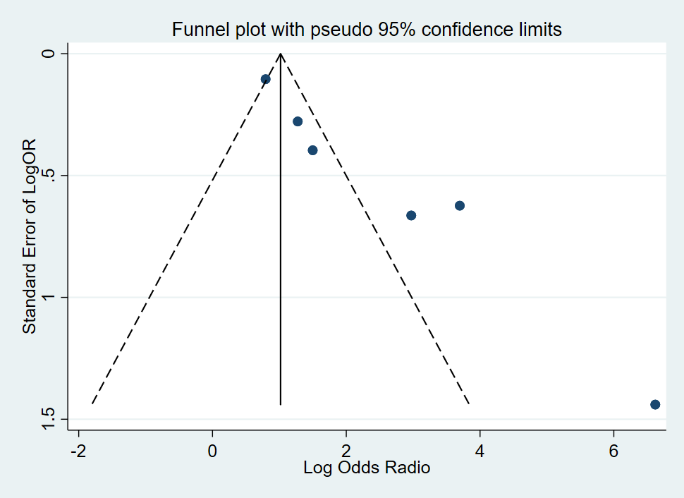


**Supplementary Fig.8:** Funnel plot corresponding to BI-RADS III+IV vs I+II in the forest plot of Fig.5.

# **Appendix H**

**Supplementary Table 2** Results of Egger’s tests

|  | **95% CI** | **P-value** |
| --- | --- | --- |
| **For case-control/cross-sectional studies** |  |  |
| BI-RADS II vs I | -8.41-5.65 | 0.657 |
| BI-RADS III vs I | -16.87-11.65 | 0.678 |
| BI-RADS IV vs I | -12.00-8.29 | 0.678 |
| BI-RADS III+IV vs I+II | -15.76-5.67 | 0.310 |
| **For case-only studies** |  |  |
| BI-RADS II vs I | -3.95-2.72 | 0.513 |
| BI-RADS III vs I | -8.02-9.13 | 0.806 |
| BI-RADS IV vs I | -16.54-13.60 | 0.715 |
| BI-RADS III+IV vs I+II | 2.46-5.69 | 0.002 |

Note: Publication bias is determined by whether the 95%CI contains 0. If 0 is not included, it indicates publication bias. Conversely, there is no publication bias.
